# Psychometric Evaluation of the Malay Version of the Childbirth Experience Questionnaire (CEQ-My)

**DOI:** 10.3390/ijerph19137644

**Published:** 2022-06-22

**Authors:** Aida Kalok, Norhani Nordin, Shalisah Sharip, Rahana Abdul Rahman, Shamsul Azhar Shah, Zaleha Abdullah Mahdy, Ixora Kamisan Atan

**Affiliations:** 1 Department of Obstetrics & Gynaecology, Faculty of Medicine, Universiti Kebangsaan Malaysia Medical Centre, National University of Malaysia, Jalan Yaacob Latif, Kuala Lumpur 56000, Malaysia; aidahani.mohdkalok@ppukm.ukm.edu.my (A.K.); honeynord@gmail.com (N.N.); drrahana@ppukm.ukm.edu.my (R.A.R.); zaleha@ppukm.ukm.edu.my (Z.A.M.); 2 Department of Psychiatry, Faculty of Medicine, Universiti Kebangsaan Malaysia Medical Centre, National University of Malaysia, Jalan Yaacob Latif, Kuala Lumpur 56000, Malaysia; shalisah@ppukm.ukm.edu.my; 3 Department of Community Health, Faculty of Medicine, Universiti Kebangsaan Malaysia Medical Centre, National University of Malaysia, Jalan Yaacob Latif, Kuala Lumpur 56000, Malaysia; drsham@ppukm.ukm.edu.my

**Keywords:** birth satisfaction, Childbirth Experience Questionnaire, childbirth, labour, postpartum

## Abstract

Negative childbirth experience may cause adverse psychological effects in postpartum mothers. The Childbirth Experience Questionnaire (CEQ) is a multidimensional tool designed to assess women’s perceptions of labour and birth. We aim to validate the Malay version of the CEQ (CEQ-My) and evaluate its psychometric properties. The previously published Malay-translated CEQ was reviewed by a panel of experts and underwent minor changes. The original visual analogue scoring (VAS) was changed to a numerical scale. The reliability and construct validity of CEQ-My was assessed using Cronbach’s alpha and exploratory analysis, respectively. Known-groups validation was conducted using the Mann–Whitney U test, whilst the inter-item correlations between CEQ-My and its subdomains were evaluated through Spearman’s correlation. The final analysis involved 246 women. The questionnaire was easy to understand and all women preferred numeric scoring to the VAS. Based on the principal component factor analysis, we deleted one item and rearranged the domain for four items. The twenty-one items CEQ-My demonstrated good reliability with Cronbach’s alpha of 0.77. Women who had spontaneous vaginal delivery demonstrated significantly greater CEQ-My scores than those who underwent operative delivery (*p* = 0.002). The domain of professional support was positively correlated to that of own capacity and participation (*p*-value of < 0.001 and 0.002, respectively). The CEQ-My is a valid and reliable instrument to assess Malaysian women’s childbirth experiences. The easy-to-use electronic version of CEQ-My will improve future research and ease data collection.

## 1. Introduction

Childbirth is one of the most significant events in a woman’s life with a potential life-long psychological effect. A positive experience may be reflected as an empowering life event connected to personal growth and self-knowledge, which influence the transition to motherhood [1]. Birth preparedness, midwifery support, intrapartum epidural, and minimal intervention during labour, contribute to a positive maternal experience [2,3]. Unplanned medical interventions such as oxytocin augmentation, operative deliveries, intrapartum complications, and neonatal care admissions are associated with maternal dissatisfaction [4].

Various maternal feelings such as control over the birth, self-esteem, fulfilment, decision making and a sense of achievement contribute to the overall experience of childbirth [3]. Women’s perception of pain is a strong predictor of the maternal childbirth experience, which is influenced by the maternal sense of control, how they cope with labour pain, and their choices of pain relief in labour [2]. A systematic review by Lally et al. demonstrated that women underestimated the pain they would experience and this expectation–experience gap may lead to less labour satisfaction [5]. Unexpected medical problems, such as emergency operative delivery or admission of an infant to neonatal care unit, as well as maternal social-related factors such as unwanted pregnancy and lack of partner’s support, were identified as predictors of negative childbirth experience [6]. A population-based study from Iceland showed that a positive attitude to childbirth during pregnancy, support from a midwife during childbirth, the use of epidural analgesia, and low intensity of pain in childbirth were amongst the strongest predictors of women’s positive childbirth experience [2].

A negative childbirth experience is related to a post-traumatic stress disorder, dysfunctional maternal-infant bonding, disruption to interpersonal relationships, and reduction in exclusive breastfeeding [3]. Negative experiences are also associated with a greater risk of postpartum depression and may adversely affect the mothers’ attitudes toward future birth, which may lead to a maternal request for caesarean delivery [7]. Assessment of women’s childbirth experiences is essential to identify women who would benefit from postpartum support and counselling. A maternal satisfaction survey is also important to understand women’s perceptions, the extent of their involvement, and the adequacy of staff-patient communications [8], which ultimately provides the quality indicator of obstetrics care and improves clinical service.

Childbirth Experience Questionnaire (CEQ), which was developed in Sweden in 2010 by Dencker et al., is a comprehensive assessment of the multidimensional nature of women’s experiences of childbirth [4]. Whilst other existing instruments for evaluating maternal experiences during labour and birth focus on isolated aspects of the childbirth experience or aggregate diverse aspects into single overall scores [9,10,11,12], the CEQ measures four major dimensions, namely *Own capacity, Professional support, Perceived safety*, and *Participation.* The CEQ consists of twenty-two items with a 4-point Likert Scale response format, except for three, which are measured using a visual analogue scale. Higher scores reflect a better and more positive childbirth experience.

The CEQ was validated among 920 primiparous women and demonstrated satisfactory Cronbach’s alpha coefficients (>0.70) in all but the participation subscale (Cronbach’s alpha 0.62). The CEQ discriminated between groups of women known to differ in childbirth experiences; for example, women with a longer duration of labour had significantly lower scores than those with shorter labour. The questionnaire has been validated in different languages, including English [13], Spanish [14], Chinese [15], Danish [16] as well as Persian [17], and is considered one of the most valid and reliable tools in its field [1]. The translated versions of CEQ were culturally adapted to maintain their validity amongst the population. The English version by Walker et al. was the first to demonstrate a good correlation between the total CEQ score and the ‘gold standard’ Maternity Survey which measures the birth experience in the United Kingdom [13]. The transcultural adaptation of CEQ to a Danish context produced a three-dimensional questionnaire [16], whilst the Chinese CEQ conformed to the original four domains but resulted in a reduction of total items to just nineteen [15]. The first Malay version of CEQ was validated by Al-kubaisi et al. in 2018 [8]. The translated questionnaire demonstrated good internal validity (Cronbach’s alpha 0.70) and good factor loadings for the majority of the items except for two. The authors concluded that the initial Malay version of CEQ is validated and reliable to the Malaysian population; however, future improvements can be made by rephrasing some of the items. Our study aimed to further validate the Malay version of CEQ by including known-groups validation as well as make it compatible with an electronic version by converting the visual analogue score to a numerical score, which will ease future data collection. Our Malay version of CEQ will be known as CEQ-My.

## 2. Materials and Methods

### 2.1. Study Design and Participants

This was a cross-sectional study involving postpartum women who delivered in the Universiti Kebangsaan Malaysia (UKM) Medical Centre, a tertiary teaching hospital in Kuala Lumpur, Malaysia. Prior ethical approval was obtained from the Universiti Kebangsaan Malaysia (UKM) Medical Research and Ethics Committee (Research Code: JEP-2019-762).

The study was conducted between April 2020 and March 2021. The inclusion criteria were women aged 18 years or older and who underwent labour at term (≥37 weeks), regardless of the mode of delivery. Exclusion criteria were women with stillbirth or abnormal foetuses, planned caesarean section, and inability to read and understand Malay.

A convenient sampling method was applied and participant recruitment was conducted amongst eligible participants during their postnatal inpatient stay. The consented women were invited to complete the electronic version of the CEQ-My on google forms using their mobile phones. The electronic version also included questions on maternal age, parity, mode of delivery as well as intrapartum analgesia. Data on labour induction, oxytocin augmentation, labour duration, NICU admission, and duration of hospital stay were collected separately by the research team.

### 2.2. Development of CEQ-My

Permission to use both the English [13] and Malay [8] versions of CEQ was obtained from the respective authors. The Malay version of CEQ was reviewed by a team of experts, which consists of midwifery staff, obstetricians, and a psychologist. Some minor changes were made to the original Malay CEQ based on the team consensus, which was felt more culturally appropriate for our population. For example, we have swapped the word ‘Bidan’ (midwife) to ‘Jururawat’ (nurse), as in our setting the word ‘Bidan’ may imply a traditional midwife.

Similar to the original version [4,13], the CEQ-My contains 22 items that measure four domains of childbirth experience: *own capacity* (8 items regarding sense of control, personal feelings during childbirth, and labour pain), *professional support* (5 items about information and midwifery care), *perceived safety* (6 items regarding sense of security and memories from childbirth), and *participation* (3 items regarding own possibilities to influence the birthing situation).

The response format for the first nineteen items is a 4-point Likert scale ranging from 1 (totally agree) to 4 (totally disagree). Reverse scoring is applied to the negatively worded statements (items 3, 5, 8, 9 and 20). The original version of CEQ uses a visual analogue score (VAS) to assess the memory of labour pain (item 20), sense of security (item 21), and control (item 22). The VAS consists of a straight line measuring 100 mm in length, of which the respondents would have to mark their response. The VAS values are then transformed to the following categorical values (0–40 mm = 1, 41–60 mm = 2, 61–80 mm = 3 and 81–100 mm = 4). The CEQ is interpreted through the mean scores of each domain. The scoring range is 1 to 4, where higher ratings reflect more positive experiences. For this study, we have converted the VAS values to an equivalent numerical scale. The participant would be asked to respond using a scale of 0 to 10 for items 20–22. We then converted the response to the following scoring (numerical response = score); 0–4 = 1, 5–6 = 2, 7–8 = 3 and 9–10 = 4.

Face validation was conducted among fifty-two postnatal women to assess the acceptability of the CEQ-My before its distribution. We randomly selected post-partum women in the obstetrics ward and conducted a face-to-face interview on the questionnaire. Each woman was shown the printed version of CEQ-My to read and answer. They were also shown both the visual analogue and numerical response for items 20–22 and were asked to indicate their preference in answering the mentioned items. All of our women had a minimum of secondary level education. All of them found the questionnaire easy to understand and acceptable to them. On average, they spent between ten to fifteen minutes completing the questionnaire and concerning items 20–22; a majority of them (fifty women) preferred to respond using the numerical score.

### 2.3. Statistical and Psychometric Analyses

Based on the recommendation by Fayers et al., we needed a sample size that is ten times the number of observed variables [18]. Taking into consideration the 10% drop-out rate due to an incomplete questionnaire, our calculated sample size was 240. The Statistical Package for Social Sciences (SPSS) version 24.0 [19] was used to analyse the study data. Data were presented as mean (standard deviation, SD) or number (percentage) for continuous and categorical variables, respectively.

The reliability of CEQ-My was assessed using Cronbach’s alpha for the whole questionnaire and each of the domains. Cronbach’s alpha > 0.7 is regarded as satisfactory [20]. Exploratory factor analysis to assess the construct validity of the CEQ-My was performed using orthogonal (varimax) rotation. The varimax rotation is chosen as it is a commonly used method to clarify the relationship among factors [21]. Bartlett’s test of sphericity and the Kaiser–Meyer–Olkin (KMO) test were used to evaluate sampling adequacy, whilst the Kaiser rule (Eigenvalue > 1.0) was applied to determine the number of dimensions to extract [22,23].

We evaluated the discriminant validity of our questionnaire through the known-groups validation method [4], in which the mean CEQ-My scores, including its domains, were compared between subgroups known to differ in key variables including parity, duration of labour, mode of delivery, labour induction, oxytocin augmentation, and intrapartum analgesia. Mann–Whitney U test was used to compare the mean scores between the groups as the CEQ-My scores for each domain were not normally distributed [22]. The inter-scale correlations between the domains and overall CEQ-My were assessed using Spearman’s correlation.

## 3. Results

### 3.1. Demographics

We distributed the electronic version of CEQ-My to 300 post-partum women and 272 completed the questionnaire online (90.7% response rate). Twenty-six incomplete responses were excluded, making the final number for analysis 246. The demographics and clinical characteristics are shown in Table 1. The duration of the active phase of labour was based on the labour room stay. Our labouring women will normally be transferred to the labour room once they develop regular contractions and reach cervical dilatation of at least 4 cm. The mean duration for the active phase of labour among our cohort was 3.7 h. We have therefore decided to use the 4 h cut-off level in the subsequent analysis of known group validation [24]. We have also divided the hospital stay duration into two groups based on the cohort’s mean of 3.4 days.

### 3.2. Exploratory Factor Analysis

All of the twenty-two items were entered in the exploratory factor analysis. The Kaiser–Meyer–Olkin (KMO) was 0.795, and Bartlett’s Test of Sphericity reached statistical significance with *p* < 0.001, supporting the sample factorability. The principal component analysis resulted in seven factors with an Eigenvalue exceeding 1. None of the items had a maximum factor loading of less than 0.30. Item 20 was excluded as it had negative loading (−0.822) and its deletion increased the overall Cronbach’s alpha to 0.767. We repeated the exploratory factor analysis using the remaining twenty-one items and presented the results in Table 2. The corrected item-total correlation values ranged between 0.090 and 0.586 (Table 2). The repeated analysis revealed six factors, which we manually grouped into four domains [25] with an Eigenvalue of 1.0 that was acceptable as well showed a good reliability score.

According to the EFA factor, each item should have a loading of at least 0.4 (moderate), with loadings of nearly 1.0 being preferred, while things with low loadings (below 0.4) should be deleted to allow for dimension reduction and therefore make the objects more stable [26]. We decided to rearrange the position for four items (5, 7, 18 and 21) as they demonstrated high factor loading in a different domain than their original [27]. We also ran Confirmatory Factor Analysis and the result are shown in Table 3. The models’ goodness-of-fit statistics revealed that none of them were overall well-fitting, with RMSEA being 0.079 and CFI and TLI relatively low.

### 3.3. Discriminant Validity

The discriminant validity of CEQ-My was assessed through known-groups validation, as shown in Table 4. Women who had spontaneous vaginal delivery scored higher in the overall CEQ-My (*p* = 0.002) as well as the subdomains of own capacity (*p* = 0.015) and participation (*p* < 0.001).

Interestingly, women who had longer hospital stays (more than 3 days) demonstrated a higher overall CEQ-My score (*p* = 0.016). Women with lower parity (*p* = 0.039), underwent oxytocin augmentation (*p* = 0.008), and had longer labour duration (*p* = 0.005) demonstrated a higher score in the participation subscale. Women who did not receive intrapartum epidural also demonstrated higher mean scores in the participation domain (*p* = 0.007). There were no significant differences in CEQ-My scores noted among different groups of women based on their age and onset of labour.

### 3.4. Internal Consistency

The Cronbach’s alpha for the 21-items CEQ-My was 0.767. Table 5 demonstrates the Cronbach’s alpha for each domain of CEQ-My and the overall score. We have also included Cronbach’s alpha of our questionnaire based on the original twenty-two items arrangement. The new CEQ-My arrangement had improved the Cronbach’s alpha for three of the domains; own capacity (0.735), professional support (0.844), and perceived safety (0.568), as well as the overall score. 

### 3.5. Inter-Scale Correlation

Table 6 depicts the inter-scale correlations among CEQ-My domains. Professional support was positively correlated to own capacity (*p* < 0.001) and participation domain (*p* = 0.005), whilst perceived safety was positively linked to own capacity (*p* < 0.001). There was a non-significant negative correlation between perceived safety and participation (*p* = 0.826), suggesting a non-linear correlation that could be contributed by many factors such as potential outliers.

## 4. Discussion

Our study had developed CEQ-My, which is a revised Malay version of CEQ. We had made some changes to the original wording, deleted one item, rearranged the domain for four items, and swapped the visual analogue scoring for a numeric scale. Assessment of psychometric properties of CEQ-My demonstrated that it is a valid and reliable measure to evaluate the childbirth experience among Malaysian women.

The Cronbach’s Alpha for CEQ-My was 0.77; which was higher than that by Al-Kubaisi et al. (0.70). Cronbach’s alpha for different versions of CEQ ranged between 0.85 to 0.90 [13,14,15,17].

Our initial exploratory factor analysis yielded seven factors. The previous Malay version of CEQ by Al-kubaisi demonstrated six components in the factor analysis, which were reduced to four following a forced analysis [8]. We excluded item 20 (*As a whole, how painful did you feel childbirth was?*) due to the negative factor loading in the EFA (−0.822), as well as the negative value for the corrected item-total correlation in the reliability analysis (−0.036). A previous study by Al-kubaisi also found a negative correlation between item 20 and the whole CEQ scale (−0.001) as well as poor factor loading of 0.24. The results from the EFA and reliability analysis on two different Malaysian cohorts suggested that item 20, in its current form, did not fit into the CEQ-My model. The cultural difference could be one of the underlying reasons. Zhu et al. demonstrated that item 20 displayed the highest ceiling effect (77.9%) and low item-total correlation (0.186). The authors suggested that item 20, which addresses pain, was not intrinsic to childbirth experiences among the Chinese women as a high rate of extreme pain was considered normal during childbirth in Chinese culture, hence supporting its exclusion from the Chinese version of CEQ [15]. We did not include the ceiling or floor effect in our statistical analysis. Further research is warranted on the effect of maternal perception of pain on the overall childbirth experience among Malaysian women.

We discovered that items 7 (*I have many positive memories from childbirth*) and 18 (*My impression of the team’s medical skills made me feel secure*) had a high factor loading in the own capacity and professional support domain, respectively; a finding similar to that of Al-Kubaisi et al. [8]. The Spanish, Chinese, and Danish versions of CEQ also demonstrated strong factor loading for item 18 in the professional support instead of its original perceived safety domain [14,15,16]. We also found that item 5 (*I was tired during labour and birth*), which was originally listed under the own capacity domain, had a high factor loading in the perceived safety domain. The relocation of these three items (items 5, 7 and 18) to their respective new domain was similar to the approach taken by Zhu et al. in the development of the Chinese version of the CEQ(CEQ-C) [15]. Zhu et al. also deleted three items (items 20, 21 and 22) as their factor analysis suggested that these items were not intrinsic to childbirth experiences among the Chinese population. We decided to relocate item 21 (*As a whole, how much control did you feel you had during childbirth?*) to the perceived safety domain as it demonstrated strong loading in the same component as item 22 *(As a whole, how secure did you feel during childbirth?)* and was strongly correlated to items 8 *(I have many negative memories from childbirth)*, *9 (Some of my memories from childbirth make me feel depressed)*, and 22. These item relocations had improved not only the internal consistency for the overall CEQ-My as well as its subscales.

Our data on known group validation were similar to the other published studies on other versions of CEQ. We found that women who had spontaneous vaginal delivery demonstrated significantly higher scores in the participation and own capacity domain, as well as the overall CEQ-My score. Non-operative delivery was also associated with higher CEQ scores among the Scandinavian, European, and Chinese populations [4,13,14,15,16].

Women with longer labour duration (more than 12 h) reported lower CEQ scores in the other version of CEQ [4,13,16]. For our known-group validation analysis, we only used the duration of the active phase of labour, which resulted in a shorter duration of labour as compared to the previously published studies. We chose to use the active phase of labour duration as the measurement was more objective than the overall labour duration, which relied on women’s recall history of the latent phase of labour.

Our study demonstrated that there was no significant difference in the overall CEQ-My score among our cohort in terms of labour duration; however, those who laboured actively for more than four hours had a significantly greater score in the participation domain. Women who had longer labour would have to decide on intrapartum analgesia, and this would be reflected in the participation domain. These women also demonstrated a non-significantly higher score in the professional support subscale, which suggests that long labour had resulted in more input from clinical staff and a higher perception of professional support. Further analysis revealed that a significant proportion of women with longer labour had also received oxytocin augmentation, which may explain the higher score in the participation domain among those in the latter group. Zhu et al. found that Chinese women who underwent labour augmentation exhibited higher scores in the professional support domain (*p* = 0.01) and participation subscale, which did not reach statistical significance [15].

Previous studies demonstrated that women with spontaneous onset of labour reported better childbirth experience [4,13,15,16] with a higher score in all the CEQ domains. Our study found no significant difference between women who differed in their labour onset. This could be due to the small number of women who underwent induction of labour in comparison to those who spontaneously laboured (36 vs. 210).

Almost half of our women received intrapartum regional analgesia. These women had lower scores in the participation domain (*p* = 0.007). Zhu et al. also found that women who used the pharmacological method of analgesia in labour had significantly lower participation scores than those who chose the non-pharmacological method of pain relief. The former also demonstrated significantly lower overall CEQ scores [15].

Studies from Spain, Denmark and China demonstrated that multiparous women reported better childbirth experiences than primiparous. Multiparas have more realistic and practical expectations due to previous childbirth experience, hence are likely to have higher labour satisfaction compared to primiparas [28,29]. First-time mothers, on the other hand, have more fear and higher expectations, which may lead to a lesser level of birth satisfaction [30,31]. These women would benefit greatly from extra support and reassurance. Although our multiparous group had a higher score than the first-time mothers, it did not reach statistical significance. The primiparous in our cohort exhibited a greater score in the participation domain (*p* = 0.039). This may be due to longer labour duration among primiparous, which heightened perception of self-involvement in the labour process, as explained previously.

Our study is the first to assess the effect of hospital stay duration on childbirth experience. The mean of hospital admission among our parturients was three days. Interestingly we found that women who had longer hospital stay reported better overall childbirth experience than those with shorter admission. These women had a higher score in their own capacity, professional support, and perceived safety domains. Inpatient hospital stay is affected by various factors, including induction of labour, prolonged labour process, operative delivery as well as a neonatal requirement for observation and treatment. Women would receive more care and input from the multidisciplinary team of health professionals, which contributed to their overall birth experience.

We found that professional support was significantly correlated to own capacity, which consisted of items relating to perceived personal expectation, control and experienced emotions, and participation domain that covered own potential to influence the birthing situation.

Women allocated continuous labour support were less likely to report negative feelings about their childbirth experience (RR 0.69, 95% CI 0.59 to 0.79) [32]. A recent local study by Adnan et al., demonstrated that women whose pregnancies were labelled as at-risk and required more attention by health workers reported greater levels of labour satisfaction than their low-risk counterparts [33]. Factors that influence maternal satisfaction toward the quality of nursing care during birth include caregiver and woman interaction, as well as woman’s involvement in the caring process [34]. Support from the health professionals was important in our cohort as it leads to a positive perception of internal emotion, but also in making choices during the birthing process. Although Spearman’s correlation between the professional and participation scales in CEQ-My was relatively weak (<0.2), the value was significant, and a further revalidation study with a larger cohort will strengthen this correlation.

Own capacity and perceived safety domains were positively correlated, most likely as both subscales dealt with the memory of childbirth. Our cohort associated positive memories with a sense of personal control (hence the relocation of item 7 to the own capacity domain), whilst negative memories were linked to a sense of security in the latter domain. Distressing recollections following experienced fear may explain the inter-correlation between fear, a sense of security, and memories of childbirth [35]. Labour is a stressful event and some women demonstrate traumatic stress symptoms such as anxiety and fear of childbirth postpartum [36,37]. Feelings of safety during childbirth also influence the memories of the delivery [38].

### Strengths and Limitations

CEQ-My is the first version of CEQ, which uses a numeric scale in place of visual analogue scoring. This resulted in a suitable electronic version that will improve data collection in the future. The majority of women nowadays, especially in urban settings such as ours, own a mobile phone; web-based questionnaires will allow more efficient data collection as well as storage. The electronic questionnaire also reduces the need for face-to-face contact, which is useful, especially during the COVID-19 pandemic.

Our version is also the first Malay-translated CEQ that has been validated for its discriminant ability. Known-groups validation revealed that CEQ-My and its domains were able to differentiate the birth experience among women who differed in their mode of delivery, labour progress, and inpatient stay.

The COVID-19 pandemic had limited our ability to conduct test-retest validation of our questionnaire. Overall, the CEQ-My demonstrated a good internal consistency; however, two of the domains (perceived safety and participation) displayed low Cronbach’s alpha values reflecting poor reliability; with the participation domain, in particular, consisting of only three items. The reason for this could be cultural and further research is needed to find additional items which will improve the internal consistency of these domains. An extension of the response scale from a four to a 5-point Likert scale will reduce the ceiling effect as well as increase the ability to detect differences between different groups. This study was conducted in a teaching hospital in the Klang Valley, which is a relatively affluent area in the country. The childbirth experience among women who delivered in a tertiary centre may be different from those who received care in a smaller district hospital. A future validation study of the improved version of CEQ-My should be multi-centric to assess its suitability in the multiethnic Malaysian population across all socioeconomic classes.

We could not assess the criterion validity for the CEQ-My as there is no ‘gold standard’ assessment of childbirth experience in Malaysia. The original CEQ was developed based on the Swedish cohort and transcultural differences are expected during the validation process. Nevertheless, CEQ-My is a valid and reliable measure of childbirth experience in the Malaysian population. It potentially may be used both in hospital settings as well as in the community as a screening instrument to identify vulnerable mothers who require counselling or support. Further research to assess the correlation between CEQ-My and postpartum depression screening tools such as Edinburgh postnatal depression scale will enhance the CEQ-My value as a screening instrument. CEQ-My can also be utilised as an audit instrument for obstetrics services, with an aim to improve future childbirth care. The six-item professional domain is useful for evaluating women’s perceptions of the care they received during labour. A future study is required to determine the population cut-off scores for CEQ-My and each domain to allow the tool to be widely used by health professionals.

## 5. Conclusions

Assessment of women’s childbirth experience is an important part of the evaluation of healthcare services. A multidimensional tool such as CEQ is essential in identifying women with negative experiences who may benefit from extra postpartum care and support. Our CEQ-My is a validated instrument that may be used to evaluate and improve obstetric care in Malaysia.

## Figures and Tables

**Table 1:** Maternal demographics and clinical characteristics.

Maternal Characteristics	Study Cohort N = 246
Age (years), mean (SD)	31.8 (4.8)
<35	168 (68.3)
≥35	78 (31.7)
Ethnicity, *n* (%)	
Malay	212 (86.2)
Chinese	24 (9.8)
Indian	8 (3.3)
Education, *n* (%)	
Primary & Secondary	76 (30.9)
Tertiary	170 (69.1)
Household income, *n* (%)	
RM 2000	8 (3.3)
RM 2000–4999	126 (51.2)
RM 5000–10,000	98 (39.8)
>RM 10,000	14 (5.7)
Parity, *n* (%)	
1	125 (50.8)
2 or more	121 (49.2)
Gestation at birth (weeks), mean (SD)	38.2(1.5)
Onset of labour, *n* (%)	
Spontaneous	210 (85.4)
Induction	36 (14.6)
Oxytocin augmentation, *n* (%)	103 (41.9)
Active phase of labour (hours), mean (SD)	3.7 (2.9)
≤4 h	162 (65.9)
>4 h	84 (34.1)
Intrapartum epidural, *n* (%)	114 (46.3)
Mode of delivery	
Spontaneous vaginal	155 (63.0)
Operative (instrumental or caesarean)	91 (37.0)
Hospital stay, mean (SD)	3.4 (1.4)
≤3 days	146 (59.3)
>3 days	100 (40.7)

SD, standard deviation; CS, caesarean section.

**Table 2:** Exploratory Factor Analysis (EFA) for 21 items of CEQ-My.

Item Number and Content		Components				
		Own Capacity	Professional Support	Perceived Safety	Participation	Corrected Item-Total Correlation
1	Labour and birth went as I had expected. *Proses melahirkan bayi adalah seperti yang telah saya jangkakan.*	0.344				0.329
2	I felt strong during labour and birth. *Saya berasa yakin dan kuat semangat sepanjang proses melahirkan bayi.*	0.645				0.510
4	I felt capable during labour and birth. *Saya berasa mampu untuk menjalani proses melahirkan bayi.*	0.511				0.471
6	I felt happy during labour and birth. *Saya berasa gembira sewaktu proses melahirkan bayi.*	0.716				0.360
7 *	I have many positive memories from childbirth. *Saya mempunyai banyak kenangan manis semasa kelahiran bayi.*	0.786				0.446
19	I felt that I handled the situation well. *Saya berasa bahawa saya telah menangani proses kelahiran dengan baik.*	0.685				0.586
13	My midwife devoted enough time to me. *Jururawat yang menjaga saya memberi perhatian yang secukupnya untuk saya.*		0.771			0.460
14	My midwife devoted enough time to my partner. *Jururawat yang menjaga saya memberi perhatian yang sewajarnya untuk pasangan saya.*		0.482			0.252
15	My midwife kept me informed about what was happening during labour and birth. *Jururawat yang menjaga saya memaklumkan kepada saya mengenai apa yang berlaku sepanjang proses melahirkan bayi.*		0.760			0.488
16	My midwife understood my needs. *Jururawat yang menjaga saya memahami keperluan saya.*		0.811			0.493
17	I felt very well cared for by my midwife. *Saya berasa saya telah dijaga dengan baik oleh jururawat yang bertugas.*		0.847			0.547
18 *	My impression of the team’s medical skills made me feel secure. *Pandangan saya terhadap kemahiran pasukan perubatan membuatkan saya rasa selamat.*		0.792			0.585
3	I felt scared during labour and birth. *Saya berasa takut sepanjang proses melahirkan bayi.*			0.745		0.077
5 *	I was tired during labour and birth. *Saya berasa letih sepanjang proses melahirkan bayi.*			0.806		0.090
8	I have many negative memories of childbirth. *Saya mempunyai banyak kenangan pahit semasa kelahiran bayi.*			0.548		0.381
9	Some of my memories from childbirth make me feel depressed. *Beberapa kenangan saya sewaktu kelahiran bayi membuat saya berasa murung.*			0.818		0.252
21 *	As a whole, how much control did you feel you had during childbirth? *Secara keseluruhan, sejauh manakah anda rasa anda dapat mengawal diri semasa melahirkan bayi?*			0.784		0.216
22	As a whole, how secure did you feel during childbirth? *Secara keseluruhan, sejauh manakah anda rasa selamat sewaktu melahirkan bayi?*			0.710		0.244
10	I felt I could have a say whether I could be up and about or lie down. *Saya berasa bebas untuk menyuarakan kehendak saya, sama ada ingin bergerak ke sana sini atau berbaring di bilik bersalin.*				0.709	0.144
11	I felt I could have a say in deciding my birthing position. *Saya berasa saya berhak membuat keputusan dalam menentukan posisi saya semasa melahirkan bayi.*				0.818	0.138
12	I felt I could have a say in the choice of pain relief. *Saya berasa saya berhak menyuarakan pendapat dalam membuat pemilihan ubat tahan sakit.*				0.379	0.136

* Item was relocated from the original domain based on factor loading in principle component analysis.

**Table 3:** 4-factor structure with maximum likelihood estimation.

Model	RMSEA	GFI	AGFI	CFI	*X^2^*	*df*
4-factor Model	0.079	0.847	0.808	0.805	462.475	184

*X*^2^ = Chi-square, df = Degrees of freedom, CFI = Comparative fit index, GFI = Goodness fit index, AGFI = Adjusted Goodness of Fit, RMSEA = Root mean squared error of approximation.

**Table 4:** Known group validation.

Group	*n*	Own Capacity	Professional Support	Perceived Safety	Participation	CEQ-My
Overall score, mean (SD)	246	3.09 (0.41)	3.34 (0.41)	2.79 (0.41)	2.72 (0.47)	3.02 (0.27)
Age						
<35	168	3.05 (0.38)	3.32 (0.40)	2.77 (0.39)	2.75 (0.46)	3.01 (0.25)
≥35	78	3.18 (0.46)	3.38 (0.44)	2.82 (0.44)	2.64 (0.49)	3.06 (0.31)
*p*-value		0.060	0.453	0.292	0.054	0.299
Parity						
1	125	3.07 (0.40)	3.29 (0.41)	2.78 (0.43)	2.77 (0.48)	3.01 (0.28)
2 or more	121	3.11 (0.42)	3.39 (0.42)	2.80 (0.38)	2.66 (0.46)	3.04 (0.26)
*p*-value		0.606	0.079	0.586	0.039	0.478
Onset of labour						
Spontaneous	210	3.08 (0.42)	3.33 (0.41)	2.80 (0.41)	2.71 (0.47)	3.02 (0.27)
Induction	36	3.19 (0.38)	3.42 (0.44)	2.74 (0.39)	2.75 (0.46)	3.06 (0.27)
*p*-value		0.095	0.298	0.273	0.678	0.222
Oxytocin augmentation						
Yes	103	3.05 (0.40)	3.39 (0.44)	2.77 (0.40)	2.82 (0.45)	3.03 (0.27)
No	143	3.13 (0.42)	3.31 (0.40)	2.80 (0.42)	2.64 (0.47)	3.02 (0.28)
*p*-value		0.118	0.209	0.382	0.008	0.878
Active phase of labour (hours)						
≤4 h	146	3.14 (0.41)	3.33 (0.41)	2.81 (0.39)	2.70 (0.47)	3.04 (0.27)
>4 h	100	3.00 (0.40)	3.37 (0.43)	2.76 (0.44)	2.75 (0.47)	3.00 (0.28)
*p*-value		0.284	0.653	0.187	0.005	0.733
Intrapartum Epidural						
Yes	114	3.07 (0.39)	3.34 (0.43)	2.74 (0.43)	2.64 (0.50)	2.99 (0.26)
No	132	3.12 (0.43)	3.34 (0.41)	2.83 (0.38)	2.78 (0.43)	3.05 (0.27)
*p*-value		0.386	0.925	0.082	0.007	0.095
Mode of delivery						
Spontaneous vaginal	155	3.15 (0.41)	3.38 (0.41)	2.83 (0.40)	2.80 (0.45)	3.07 (0.26)
Operative delivery	91	3.00 (0.40)	3.28 (0.41)	2.73 (0.42)	2.60 (0.47)	2.94 (0.26)
*p*-value		0.015	0.134	0.077	<0.001	0.002
Hospital stay						
≤3 days	146	3.07 (0.40)	3.31 (0.39)	2.79 (0.41)	2.71 (0.45)	3.01 (0.26)
>3 days	100	3.12 (0.42)	3.38 (0.44)	2.80 (0.41)	2.72 (0.51)	3.04 (0.28)
*p*-value		0.061	0.051	0.302	0.438	0.016

Data presented as mean unless otherwise stated. Mann–Whitney U test was used as the scores were not normally distributed. SD, standard deviation.

**Table 5:** Cronbach’s Alpha for the domains of CEQ-My and the overall scale.

	Original CEQ Order		CEQ-My	
	No of Items	Cronbach Alpha	No of Items	Cronbach Alpha
Own capacity	8	0.430	6	0.735
Professional support	5	0.814	6	0.844
Perceived safety	6	0.551	6	0.568
Participation	3	0.488	3	0.488
Total score	22	0.747	21	0.767

**Table 6:** Inter-scale correlations between the CEQ-My domains.

Domain	Own Capacity	Professional Support	Perceived Safety	Participation
Own capacity (*p*-value)	1	0.471 * (<0.001)	0.282 * (<0.001)	0.089 (0.163)
Professional support (*p*-value)	0.471 * (<0.001)	1	0.052 (0.413)	0.178 * (0.005)
Perceived safety (*p*-value)	0.282 * (<0.001)	0.052 (0.413)	1	−0.014 (0.826)
Participation (*p* value)	0.089 (0.163)	0.178 * (0.005)	−0.014 (0.826)	1

* Correlation is significant at the 0.01 level (2-tailed).

## Data Availability

The data presented in this study are available on request from the corresponding author.
